# Zinc Fingers and Homeobox Family in Cancer: A Double-Edged Sword

**DOI:** 10.3390/ijms231911167

**Published:** 2022-09-22

**Authors:** Yonghua Bao, Haifeng Zhang, Zhixue Han, Yongchen Guo, Wancai Yang

**Affiliations:** 1 Department of Pathology, Mudanjiang Medical University, Mudanjiang 157011, China; 2 Department of Immunology, Mudanjiang Medical University, Mudanjiang 157011, China; 3 Comprehensive Cancer Center, The Ohio State University, Columbus, OH 43210, USA

**Keywords:** zinc fingers, homeoboxes, ZHX1, ZHX2, ZHX3, cancer

## Abstract

The zinc fingers and homeobox (ZHX) family includes ZHX1, ZHX2, and ZHX3, and their proteins have similar unique structures, containing two C2H2-type zinc finger motifs and four or five HOX-like homeodomains. The members of the ZHX family can form homodimers or heterodimers with each other or with a subunit of nuclear factor Y. Previous studies have suggested that ZHXs can function as positive or negative transcriptional regulators. Recent studies have further revealed their biological functions and underlying mechanisms in cancers. This review summarized the advances of ZHX-mediated functions, including tumor-suppressive and oncogenic functions in cancer formation and progression, the molecular mechanisms, and regulatory functions, such as cancer cell proliferation, migration, invasion, and metastasis. Moreover, the differential expression levels and their association with good or poor outcomes in patients with various malignancies and differential responses to chemotherapy exert opposite functions of oncogene or tumor suppressors. Therefore, the ZHXs act as a double-edged sword in cancers.

## 1. Introduction

The zinc fingers and homeobox (ZHX) family have three members, including ZHX1–3 [1,2,3,4]. They share similar gene and protein structures [5]. ZHX family members contain a unique protein structure, two C2-H2 (Cys-Xaa2-Cys-Xaa12-His-Xaa4-His) zinc finger motifs and four or five HOX-like homeodomains (HDs). 

The humansZHX1 and ZHX2 are located on chromosome 8 [1,4], and ZHX3 is located on chromosome 20 [6]. ZHX1–3 can constitute homodimers or heterodimers and can interact with the alpha subunit of nuclear factor Y (NFYA) to repress transcription [1,2,3,6,7,8]. 

### 1.1. ZHX1 Gene and Biological Functions

ZHX1 was initially identified from a murine bone marrow stromal cell [9]. Human ZHX1 was identified by screening a human liver cDNA library for NFY-interacting protein isolation [4]. The ZHX1 gene of humans is located in Chr8q24.13, spanning approximately 27 kb. ZHX1 has eight transcripts, including two main ones, 4.5 kilobases (kb) and 5 kb, respectively. These two transcripts are expressed ubiquitously, although the latter is more abundant in most tissues [4]. 

Human ZHX1 contains 873 amino acid residues and is structurally composed of two C2-H2 motifs and five homeodomains [4]. This is why it is classified as a zinc finger of homeodomain transcription factor. ZHX1 forms homodimers or heterodimers through the homeodomain 1 region (amino acids 272–432). ZHX1 functions as a transcriptional repressor via an acidic region (amino acids 831–873). Achieving complete inhibitory activity requires dimerization [7].

ZHX1 proteins of humans and mice possess 91% amino acid similarity [8]. The ZHX1 gene of mice is located in chromosome 15, approximately 29 kb in length [2]. ZHX1 mRNA is widely expressed in adult mice, with a high expression in the brain and lower levels in the liver and kidney [9]. It has been reported that the mouse ZHX1 gene expression could be induced by IL-2 in CTLL-2 cells [10]. 

Besides interacting with NFYA, ZHX1 binds DNA methyltransferase 3B [11] and the transcriptional corepressor BS69 to induce repression activity [12].

### 1.2. ZHX2 and Biological Functions

Human ZHX2 was cloned as a novel ubiquitous transcription factor and a ZHX1-interacting protein from a yeast two-hybrid screen on a size-fractionated brain cDNA library [1]. The ZHX2 gene is located on Chr8q24.13, spanning approximately 193 kb. This gene has two transcripts (splice variants) coding 837aa and 166aa. ZHX2 mRNA is expressed in various tissues, with the highest levels in the ovaries, and sequentially lower levels in the lung, heart, kidney, brain, and liver. The pancreas, spleen, testis, and skeletal muscle have intermediate expression levels. A genome-wide association study has demonstrated that individuals with the ZHX2 polymorphism G779A have a strong response to the smallpox vaccine [13].

The mouse ZHX2 gene encodes an 836aa protein, having 87% amino acid identify with the human ZHX2 protein [14]. ZHX2 contains two C2-H2 zinc finger motifs and five HDs. This region, called the P domain, is rich in proline and resides between HD1 and HD2. ZHX2 forms a homodimer with itself or a heterodimer with ZHX1 at the region containing HD1. In addition, ZHX2 can interact with NFYA at the region between HD1 and HD2. Further studies have revealed that nuclear ZHX2, not cytoplasmic ZHX2, acts as a transcriptional repressor. The repressor domain resides in a region between the HD1 and P domains [1,3]. 

ZHX2 was previously thought to suppress gene transcription. However, in vitro and in vivo studies showed that ZHX2 positively regulates major urinary proteins’ (Mups) gene expression [15].

### 1.3. ZHX3 and Biological Functions

The human ZHX3 gene is located on Chr20q12, spanning approximately 139 kb, and has 21 transcripts (splice variants). ZHX3 has 956 amino acid residues and, like ZHX1, contains two C2-H2 motifs and five HDs [6]. ZHX3 forms a heterodimer with ZHX1 and interacts with NFYA. The pleiotropic HD1 region is responsible for the dimerization with ZHX1, interaction with NFYA, and repressor function. In addition, two nuclear localization signals were mapped to the N-terminus at zinc fingers 1 and the HD2 region. Moreover, protein–protein interaction assays showed that ZHX3 formed a heterodimer with ZHX2 via a region containing HD1. In mice, there are three ZHX3 transcripts of 9.5 kb, 6.5 kb, and 4.4 kb in length, which are ubiquitously and differentially expressed. Functional studies have revealed that nuclear ZHX3 is a ubiquitous transcriptional repressor and functions as a dimer [8,16].

Accumulating evidence has demonstrated that ZHX family members play important roles in cell development, differentiation, and various cancers [17,18,19,20,21,22]. The aberrant expression and dysfunction of ZHXs are associated with the occurrence and progression of various diseases. These include hematological, neurological, and glomerular diseases, as well as carcinogenesis and its progression [22]. The expression levels are also linked to the outcomes of several malignancies. Interestingly, the biological functions of ZHXs in cancers are not consistent. For example, ZHXs act as tumor suppressors or oncogenes in different types of cancers. The expression levels of ZHXs are also associated with poor or good outcomes in patients with various malignancies, and act as a “double-edged sword”. This review focuses on the clinical significance of the oncogenic or tumor-suppressive functions of ZHX family members in cancers.

## 2. Oncogenic or Tumor-Suppressive Functions of ZHXs in Cancers

### 2.1. ZHX1 and Cancer

**Figure 1:**
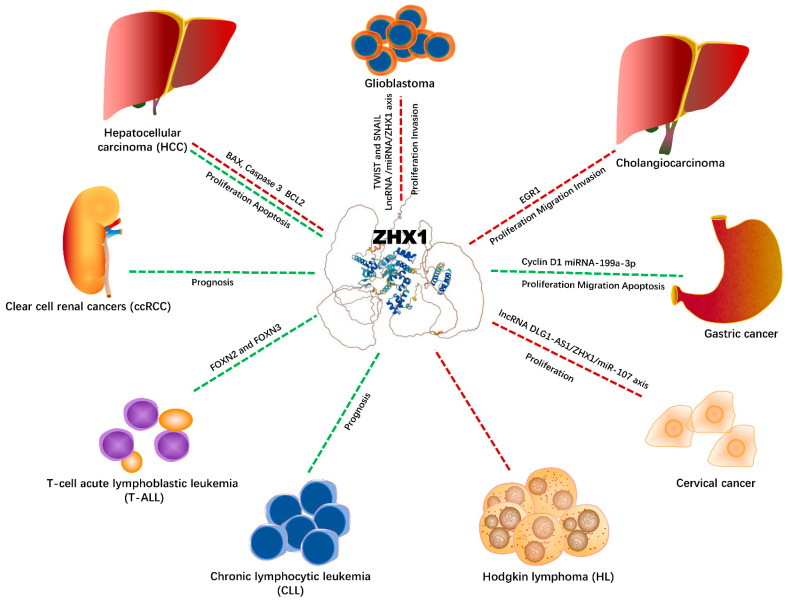
ZHX1 and cancers: biological functions and potential signaling networking.

ZHX1 has recently been involved in the tumorigenesis of various tumors. The regulation of ZHX1 expression, its clinical significance, and role in cancer is controversial. ZHX1 acts as an oncogene or tumor suppressor in different types of cancer (Table 1). ZHX1 contributes to cancer cell proliferation, mobility, migration, and invasion (Figure 1). In glioblastoma [23], the messenger RNA and protein expression of ZHX1 are increased compared with normal brain tissue, and ZHX1 overexpression was associated with short overall survival. In vitro, the knockdown of ZHX1 expression suppressed cell proliferation, mobility, migration, and invasion in glioblastoma. However, increasing the expression of ZHX1 enhanced malignant features, which are linked to the regulation of TWIST and SNAIL, both of which are transcriptional factors for the epithelial–mesenchymal transition (EMT) and metastasis. 

It has been reported that long non-coding RNA (lncRNA), microRNA (miRNA), and ZHX1 participate in tumor initiation and progression. In vitro and in vivo studies have shown that LncRNA MALAT1 facilitates glioblastoma cell proliferation and progression by reducing miR-199a to promote ZHX1 expression. The LncRNA MALAT1-elevated expression is associated with the poor prognosis of glioblastoma patients [24]. Another study on glioma also showed that ZHX1 is an oncogene, and it was identified as a target gene of miR-23b-3p. LncRNA SNHG17 can absorb miR-23b-3p like a sponge to elevate ZHX1expression; therefore, increased SNHG17 and ZHX1 expressions and the reduced miR-23b-3p expression were found in glioma tissues. Oncogenic SNHG17 improves cell proliferation, migration, and invasion in glioma [25]. In addition, lncRNA LINC01140 promote the development of glioma by downregulating miR-199a-3p expression and indirectly upregulating ZHX1 expression [26]. These findings strongly suggest that targeting the lncRNA/miRNA/ZHX1 pathway might provide a new therapeutic strategy for tumors of the nervous system.

Another study has demonstrated the oncogenic roles of lncRNA DLG1-AS1/miR-107/ZHX1 axis in cervical cancer [27]. lncRNA DLG1-AS1 was remarkably overexpressed in cervical cancer tissues, and the cervical cancer patients with a high DLG1-AS1 expression have worse outcomes. Manipulating gene expression in vitro showed that the knockdown of the DLG1-AS1 gene inhibited the proliferation of cervical cancer cells. Mechanical studies showed that the inhibition of miR-107 abrogated DLG1-AS1-mediated ZHX1 expression via competitive binding between ZHX1 and miR-107. Vice versa, increasing the expression of miR-107 rescued the role of DLG1-AS1 in cervical cancer cell proliferation, indicating that the oncogenic effects of DLG-AS in cervical cancer are miR-107/ZHX1-dependent. ZHX1 also acts as an oncogene in cholangiocarcinoma [28]. Immunohistochemical staining analysis revealed that ZHX1 is amplified and overexpressed in cholangiocarcinoma tissues. In vitro studies showed that overexpressing ZHX1 facilitated cholangiocarcinoma cell proliferation, migration, and invasion. Conversely, ZHX1 knockdown using specific small interfering RNAs decreased malignant characteristics. Mechanistic studies showed that ZHX1-mediated tumor promotion might be partially associated with early growth response 1 (EGR1).

In contrast, ZHX1 exerts tumor-suppressive functions in gastric cancer [29], in which ZHX1 expression was reduced. Interestingly, the reduced nuclear expression of ZHX1 was closely related to a larger tumor size, poorer differentiation, advanced TNM stages, and a deeper invasion. However, these features were not correlated with lymph node metastasis. In cultured gastric cancer cells, stable transfection with plasmids overexpressing ZHX1 significantly promoted apoptosis, and inhibited cell proliferation and migration. In nude mice, the ZHX1 overexpression inhibited the tumor growth that was associated with cell cycle arrest and a repressed expression of cyclin D1.

Another study on gastric cancers reported by Wang et al. also showed that ZHX1 was a tumor suppressor and a downstream target of tumor promoter miRNA-199a-3p [30]. MiR-199a-3p expression was increased in gastric cancer tissues and gastric cancer cell lines. The study also demonstrated that miR-199a-3p could negatively regulate ZHX1 gene expression in gastric cancer cells. Therefore, increasing the expression of miR-199a-3p inhibits ZHX1 expression, but reducing miR-199a-3p expression promotes ZHX1 expression. Furthermore, restoring ZHX1 expression inhibits cancer cell proliferation in vitro.

A more recent study indicated that an increased ZHX1 expression was associated with a better or worse prognosis for patients with gastric cancer in the clinical stages, including lymph node metastasis or distant metastasis, and surgical therapy [31]. For instance, a lower ZHX1 expression was related to a better prognosis of patients without lymph node metastasis, whereas a higher ZHX1 expression was related to a better prognosis of patients without distant metastasis. Furthermore, a higher ZHX1 expression was associated with a better prognosis of the patients who received surgery therapy and in the patients whose cancer showed poor differentiation.

A study on clear cell renal cancers (ccRCC) [18] based on an online database showed that both ZHX1 and ZHX3 expression levels were downregulated, while ZHX2 was upregulated. In particular, the ZHX1 and ZHX3 expression levels were remarkably lower, but ZHX2 expression was higher in the advanced stages compared with the early stages. In addition, ZHX1 and ZHX3 expression levels were associated with the T stage, while ZHX1 expression was associated with the M stage. These results suggest that a lower expression of ZHX1 and ZHX3 is related to the progression of clear cell renal cancers. Furthermore, Kaplan–Meier and multivariate regression analysis showed that a reduction in ZHX1 mRNA expression is associated with worse survival [18]. Altogether, these findings indicate that ZHX1 is a tumor suppressor in renal cancer.

In hepatocellular carcinoma (HCC), ZHX1 has also been reported as a tumor suppressor. One study [32] detected a decreased ZHX1 expression from a HCC tumor and cell sample. In vitro studies have indicated that an increased expression of ZHX1 suppresses HCC SMMC-7721 cell proliferation. Another study also showed the tumor-suppressive roles of ZHX1 in HCC, in which miR-199a-3p targeting the ZHX1/PUMA signal was reported to inhibit tumorigenesis. In this case, miR-199a-3p inhibited HCC HepG2 cell growth in vitro and induced apoptosis by upregulating the expression of ZHX1 and PUMA [33]. In contrast, the knockdown of ZHX1 or PUMA reversed miR-199a-3p-mediated tumor inhibition roles, as determined by a mechanistic investigation, suggesting that the miR-199a-3p/ZHX1/PUMA signaling could be linked to BCL2, BCL2-associated X, and cleaved caspase 3.

The prognostic impact of ZHX1 and ZHX2 in chronic lymphocytic leukemia (CLL) has recently been reported [34]. CLL patients with reduced ZHX1 and ZHX2 expression have a worse prognosis. In addition, the accumulation of chromosomal abnormalities was negatively associated with ZHX1 and ZHX2 expression in CLL patients.

More studies support the tumor-suppressive roles of ZHX1 in T-cell acute lymphoblastic leukemia (T-ALL), showing that ZHX1 was significantly reduced in T-ALL cells; moreover, other tumor suppressors, such as FOXN2 and FOXN3, were also concurrently downregulated in the T-ALL cell lines, in which both FOXN2 and FOXN3 formed a regulator network and directly activated the transcription of ZHX1 [35]. Furthermore, the physiological expression profile of normal hematopoietic cells revealed that ZHX1 was highly expressed in T-cells and was lower in B-cells, suggesting biased functions of ZHX1 in lymphopoiesis [36]. Lastly, the Hodgkin lymphoma cell lines that showed no expression of orthodenticle homeobox 1 and 2 (OTX1/2) (e.g., OTX 1 and 2 negative) overexpressed ZHX1, correlating with the genomic amplification of the 8q24 locus, supporting the oncogenic potential of ZHX1 in Hodgkin lymphoma [36].

### 2.2. ZHX2 and Cancer

Several studies have demonstrated the tumor-suppressor activities of ZHX2 in hepatocellular carcinoma (HCC) and other malignancies (Table 2), but accumulating evidence has also shown that ZHX2 plays oncogenic roles in carcinogenesis and progression in various cancers (Table 2).

**Figure 2:**
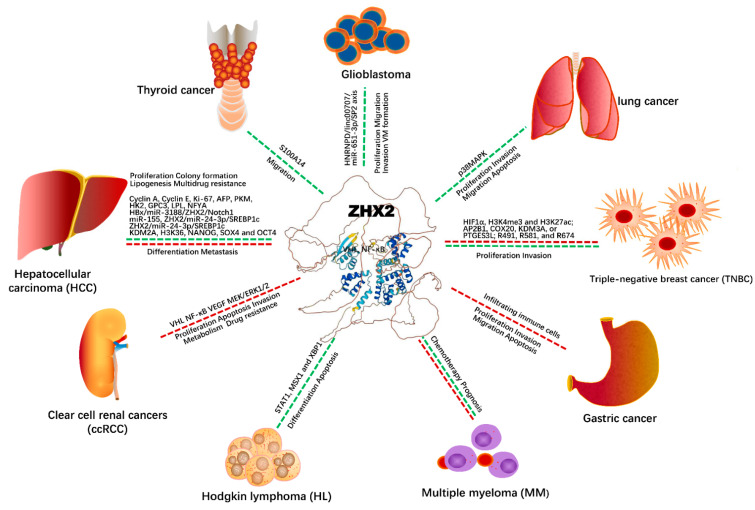
ZHX2 and cancers: biological functions and potential signaling networking.

ZHX2-involved tumor occurrence, development, progression in various tumors, and potential molecular mechanisms are summarized in Figure 2. Tumor-suppressive functions of ZHX2 were supported by the following studies, with most from hepatocellular carcinoma (HCC). Elevating ZHX2 expression can suppress HCC cell multiplication, colony formation, and mice tumor size. Conversely, reducing ZHX2 expression by siRNA significantly enhanced cell proliferation and colony formation [37]. Mechanistic studies demonstrated that tumor inhibition was through the suppression of Cyclins A and E via a direct binding between ZHX2 and the promoter domain of Cyclins A and E. The inverse correlation between ZHX2 and Cyclins A and E was further detected from liver cancer tissues. The overexpression of ZHX2, including nuclei and the cytoplasm, did not differ in tumor tissues and adjacent non-tumor tissues. Interestingly, a lower nuclear ZHX2 expression and a higher cytoplasmic ZHX2 expression were found in HCC tissues, but a higher nuclear ZHX2 expression and lower cytoplasmic ZHX2 expression were found in adjacent non-tumor tissue. This suggests that a reduced nuclear expression and increased cytoplastic expression of ZHX2 could play roles in hepatocarcinogenesis. This was further supported by cellular fragment analysis, showing that the low nuclear expression of ZHX2 was correlated with a high Cyclins A and E and Ki-67 expression level. Moreover, the patients with reduced ZHX2 nuclear expression have poor tumor differentiation, larger sizes, and a significantly shorter survival time [37]. This indicated the critical tumor-inhibitory roles of nuclear ZHX2 in HCC progression. Epigenetic analysis results have revealed that a reduced expression of ZHX2 was due to the hypermethylation of the ZHX2 promoter [38].

Figure 2 A previous study demonstrated that the ZHX2 protein was involved in the postnatal repression of alpha fetoprotein (AFP) in mice [39]. Other studies have shown that ZHX2 expression in HCC was inversely correlated with serum AFP levels in HCC patients [14], suggesting a repressive role of ZHX2 on AFP. This was supported by in vitro experiments, showing that ZHX2 overexpression significantly decreased AFP secretion in HCC cells and that reducing the expression of ZHX2 restored APF expression in LO2 and SMMC7721 cells. Luciferase-based assays revealed that the repression of AFP by ZHX2 is regulated by the AFP promoter and requires intact HNF1 binding sites [40]. Besides the repression of AFP, ZHX2 also repressed the cancer biomarkers pyruvate kinase M1/2 (PKM) and hexokinase 2 (HK2) in HCC [16]. Moreover, ZHX2 suppressed oncogenic activation of glypican 3 (GPC3) in HCC [41]. Special attention has been given to GPC3 because of its diagnostic potential in HCC [61]. However, ZHX2 expression was negatively associated with GPC3 from HCC tissues and in cultured liver cell lines. In these studies, the increased expression of ZHX2 markedly reduced GPC3 expression, while the downregulation of ZHX2 by small molecules elevated GPC3 expression levels in vitro-cultured cells. Mechanistic studies have revealed that the suppressive action of ZHX2 is achieved through binding to the GPC3 promoter [41].

Epidemiology and clinical studies have demonstrated that virus-related hepatocellular carcinoma, particularly hepatitis B virus (HBV)-related HCC, is one of the main types of HCC [62]. During carcinogenesis of HCC, ZHX2 also plays a tumor-inhibitory role through dysregulating HBV X protein (HBx). In turn, HBx plays an important part in HBV-related HCC, activating miR-3188 and Notch signaling via CREB, and then inhibiting ZHX2 expression [42]. Conversely, the interaction between ZHX2 and NFYA can reduce Notch1. It has been reported that miR-3188 plays an oncogenic role and is highly expressed in HBV transgenic mice liver and HepG2.215 cells. Knockdown of miR-3188 inhibits cell proliferation, migration, invasion, and mouse tumor growth [42]. Thus, HBx-miR-3188-ZHX2-Notch1 is considered as the major signaling pathway during carcinogenesis and development in HBV-associated HCC. Song et al. also reported that ZHX2 is an HBV-related tumor suppressor gene in HCC [43]. ZHX2 expression is significantly decreased in tumor tissues of HBV-positive HCC and livers of HBV-transgenic mice. Further studies confirmed that ZHX2 expression levels and tumor-suppressive activities were inhibited by HBV-encoded proteins, particularly HBx [43]. Mechanistic studies showed that ZHX2 expression levels were affected by miR-155 via its seed sites in the ZHX2 3UTR. In fact, miR-155 levels were significantly increased in HBx-overexpressing HCC cell lines, HBV-positive HCC tissues, and HBV-transgenic mouse livers. Interestingly, ZHX2 levels were upregulated when miRNA-155 levels were blocked in vitro [43]. These findings suggest that HBV-mediated HCC-promoting properties could be attributed to the miR-155-induced silencing of ZHX2, and they suggest a novel therapy for HBV-related HCC by targeting the miR-155 pathway. 

As an HCC-associated tumor suppressor, ZHX2 is found to be hepatoprotective by reducing liver lipid levels in a high-fat diet-fed rodent model [63]. In addition, ZHX2 could suppress lipoprotein lipase and indirectly prevent HCC formation [64]. It is known that lipogenesis plays an important role in carcinogenesis and the development of HCC. An experimental study has shown that the overexpression of ZHX2 in HCC cells significantly inhibited de novo lipogenesis [44]. ZHX2 expression was inversely correlated with the expression of SREBP1c. Further studies have revealed that ZHX2 inhibited liver tumorigenesis and development by inhibiting SREBP1c, which regulates de novo lipogenesis. The therapeutic strategy provided additional evidence showing that fatostatin, as a SREBP1c suppressor, attenuated liver tumorigenesis in Zhx2 conditional deletion in the liver of mouse models. Mechanistically, ZHX2 upregulated miR-24–3p expression at transcription levels, further leading to the degradation of SREBP1c targeted by miR-24–3p. Conclusively, these data provided a novel mechanism for ZHX2 inhibiting HCC progression, and targeting the ZHX2/SREBP1c axis could be a novel treatment method of liver cancer.

Numerous studies have proved that non-alcoholic fatty liver disease (NAFLD) is closely associated with hepatocellular carcinoma (HCC). ZHX2 was found to be decreased in NAFLD-HCC liver tissue [45]. This study showed that increasing ZHX2 expression disturbed lipid homeostasis and hepatocytes’ lipid deposition and prevented exogenous lipids uptake via the suppression of lipid lipase (LPL), resulting in the inhibition of HCC cell proliferation. Vice versa, increasing LPL expression in HCC cells could reverse ZHX2-mediated cell proliferation inhibition, tumor growth in a xenograft mouse model, lipid metabolism, and tumor formation in mouse liver. Moreover, in an HCC cohort study, immunohistochemical staining demonstrated a negative correlation between ZHX2 and LPL expression. Taken together, ZHX2 protects hepatocytes from cell growth and NAFLD-HCC progression through regulating lipid deposition and the transcriptional repression of LPL [45].

ZHX2 expression levels have been significantly reduced in liver cancer stem cells (CSCs) from different origins [46]. Manipulating the deficiency of ZHX2 could enhance tumor progression and liver CSC stemness. Elevating ZHX2 expression attenuated liver CSCs transformation from the initiation of tumor formation, self-renewal, and sorafenib-resistance. Mechanical studies have demonstrated that ZHX2 suppresses liver CSCs via epigenetic regulation, that is, by suppressing the KDM2A-related demethylation of histone H3 lysine 36 (H3K36), because the H3K36 promoter has multiple binding sites of stemness-associated transcription factors, including NANOG, SOX4, and OCT4. Clinical studies have found that a lower expression of ZHX2 and a higher KDM2A expression were associated with a shorter survival time, which was linked to the transcriptionally repressing KDM2A by ZHX2 in HCC. These findings have improved our understanding of the molecular mechanisms of HCC relapse and drug resistance [46].

Therapeutic studies have also demonstrated that ZHX2 improved the cytotoxicity of chemotherapeutic drugs by inhibiting multidrug resistance 1 (MDR1) via an interaction with NFYA in liver tumor cells [48]. Firstly, an inverse correlation of ZHX2 and MDR1 expression levels was observed in HCC tissues by immunohistochemical staining. Secondly, luciferase reporter assays showed that ZHX2 repressed the promoter activity of the MDR1. However, the knockdown of NFYA or mutation of the NFY binding site eliminated the ZHX2-mediated repression of MDR1 at a transcriptional level. This suggests that ZHX2 was interacting with NFYA, thus, reducing NFY binding to the MDR1 promoter. 

However, it has been reported that the ZHX2 protein was overexpressed in liver cancer tissues, and that its upregulation expression was related to poor differentiation and cancer metastasis [47], suggesting an oncogene role of ZHX2 in HCC.

In lung cancer, ZHX2 exhibits tumor suppressor functions. For example, ZHX2 expression was significantly reduced in the human lung cancer cell lines [49]. Compared to the control groups, cell growth, moving, and invasion were dramatically inhibited by ZHX2, in which cell apoptosis and apoptosis-related proteins were increased. In addition, ZHX2 inhibited tumor growth in terms of the reductions in growth rate, tumor size and weight, and PCNA-positive cell numbers. Mechanically, ZHX2 acts as a tumor suppressor in lung cancer by inhibiting the p38MAPK signaling pathway.

In glioma cells, it has been reported that ZHX2 interacts with HNRNPD and further regulates vasculogenic mimicry (VM) formation through the linc00707/miR-651–3p/SP2 pathway [50]. The expression levels of ZHX2 and miR-651–3p were remarkedly reduced, while HNRNPD, linc00707, and specific protein 2 (SP2) expression were remarkedly increased in glioma. Moreover, increasing the ZHX2 and miR-651–3p expression or decreasing the expression of HNRNPD, linc00707, and SP2 suppressed glioma cells’ proliferation, migration, invasion, and VM formation. Interestingly, by reducing the HNRNPD expression, it caused an increase in ZHX2 mRNA stability, and ZHX2 negatively regulated the linc00707 expression by binding to its promoter region. Firstly, linc00707 binds with miR-651–3p, and the latter binds to the SP2 mRNA 3′ untranslated region (3′UTR) and negatively regulates its expression. Then, SP2 binds to the promoter regions of MMP2, MMP9, and VE-cadherin, which are VM formation-related proteins, and therefore, play a role in enhancing transcription for the regulation of glioma cell VM formation.

It was also reported that ZHX2 inhibited the progression of thyroid cancer [51]. The decreased ZHX2 expression in thyroid cancer tissues was correlated with poor outcomes. The knockdown of ZHX2 significantly promoted thyroid cancer cell migration. S100 calcium-binding protein (S100A14) was highly expressed in human thyroid cancers, showing a negative correlation with ZHX2. Mechanistically, ZHX2 binds to the promoter region of the S100 to decrease its transcription. The inhibition of S100A14 attenuated thyroid cancer cell metastasis induced by ZHX2 knockdown in cultured cells and in animal models. 

Multiple myeloma (MM) is an incurable hematological malignancy. Compared to the low-risk or indolent disease, ZHX2 expression was remarkably reduced in the high-risk or active disease. The multiple myeloma patients with lower ZHX2 expression have worse outcomes [54]. Other studies have also reported that increasing the ZHX2 expression could improve the response to high-dose chemotherapy in multiple myeloma [52,53]. In contrast, another study by Jiang et al. reported that multiple myeloma patients with a higher ZHX2 expression showed poorer clinical outcomes, and the knockdown of ZHX2 in cancer cells caused MM cells to be more sensitive to Bortezomib (BTZ) by regulating the nuclear translocation of NF-κB, also affecting NF-κB and the corresponding target genes’ expression at the mRNA levels [55].

ZHX2 has also been reported as a tumor suppressor in Hodgkin lymphoma, where the recurrent breakpoint at 8q24 targets ZHX2. This aberration broke the far upstream activation elements of ZHX2 and decreased ZHX2 expression [56]. The gene expression profiling results indicated the regulating function of ZHX2 on differentiation and apoptosis. STAT1 (signal transducer and activation of transcription 1) and several STAT1 target genes were included, suggesting that ZHX2 functions as a tumor suppressor in Hodgkin lymphoma [56]. Further studies demonstrated the transcriptional deregulation of ZHX2 in B cell malignancies. Two transcription factors, homeodomain protein msh homeobox 1 (MSX1) and bZIP protein X-box binding protein 1 (XBP1), were shown to directly regulate ZHX2 expression. Multiple mechanisms might be involved in the suppression of ZHX2 expression in Hodgkin lymphoma cell lines. These include the loss of activation elements of ZHX2 upstream and a decreased expression of activators MSX1 and XBP1 [57]. 

ZHX2 can drive tumorigenesis in clear cell renal cell carcinoma (ccRCC). The loss of VHL usually upregulates ZHX2 levels, in particular, the nuclear expression of ZHX2 in ccRCC tumors. Mechanistic studies showed that VHL-upregulating ZHX2 was achieved through prolyl hydroxylation and proteasomal degradation signaling, supported by an experiment showing that the inhibition of prolyl hydroxylation and the degradation of proteasome could increase ZHX2 expression. The engineered deletion of ZHX2 caused decreased anti-apoptosis-associated multiple gene expression and inhibited cancer cell growth, metastasis, and metabolism. Moreover, ZHX2 promoted NF-κB activation and drove renal carcinogenesis. These studies reveal the oncogenic functions of ZHX2 in renal cancer and uncover a potential therapeutic target for ccRCC [21]. The ccRCC study of Zhu et al. found an additional mechanism. ZHX2-driven cell proliferation and migration were achieved though the activation of the MEK/ERK1/2 signaling pathway and the increasing VEGF expression, and led to Sunitinib resistance through regulating self-protective autophagy, providing a new insight for advanced ccRCC treatment [58].

Triple-negative breast cancer (TNBC) belongs to a more aggressive breast cancer subtype with a higher mortality rate; therefore, it is an urgent requirement to find a novel treatment strategy. Studies have demonstrated that ZHX2 is amplified or increased in TNBC patient tissues. Functional studies showed that manipulating the deletion of ZHX2 inhibits TNBC tumor growth and metastasis. Chromosomal immunoprecipitation sequencing results have shown that ZHX2 binds and transcriptionally activates HIF1α (hypoxia-inducible factor alpha), finally promoting gene expression. AP2B1, COX20, KDM3A, and PTGES3L were regulated by both ZHX2 and HIF1α—their overexpression could partially rescue ZHX2 depletion-caused cell growth in TNBC cells. These findings strongly suggest that these target genes synergistically help the oncogene function of ZHX2. Genomic studies further showed that three residues (R491, R581, and R674) on ZHX2 are important for ZHX2 to exert its transcriptional activity. It was suggested that ZHX2 acts as an oncogene via activating the HIF1α pathway in TNBC, and ZHX could be used as a novel therapeutic target for TNBC [59].

It has been reported that ZHX2 forms heteromeric complexes with ZHX3 [8,16]. Their effect on breast cancer prognosis is similar. Both ZHX2 and ZHX3 expressions were remarkably reduced in TNBC tissues, and a high mRNA expression of ZHX2 and ZHX3 were closely associated with a better prognosis of breast cancer patients, especially in luminal A subtype breast cancer. In addition, the mRNA expression of ZHX2 and ZHX3 were also positively associated with the estrogen receptor and progesterone receptor, but ZHX2 mRNA expression was inversely correlated with HER2 in breast cancers [19].

In gastric cancer (GC), ZHX2 also exerts an oncogenic function [60]. An increase in ZHX2 expression was closely correlated with clinical characteristics; for instance, a higher ZHX2 expression predicted worse outcomes and immune infiltrating. An in vitro experimental study showed that ZHX2 overexpression can facilitate gastric cancer cell proliferation and migration and can suppress cell apoptosis. These findings suggest ZHX2 as a prognostic biomarker, and immune infiltration could be associated with the effect of ZHX2 on gastric cancer.

By contrast, gastric cancer patients with a decreased ZHX2 and ZHX3 mRNA expression showed better outcomes [31]. Reduced ZHX2 and ZHX3 expression was remarkably correlated with a longer survival time in the subgroups of GC patients without lymph node metastases or distant metastasis, suggesting that the low expression of ZHX2 and ZHX3 predicts a better prognosis for early-stage gastric cancer patients. Subgroup analyses also found that the downregulation of ZHX2 was predictive of an improved survival time in HER2-positive gastric cancer patients, but not in HER2-negative gastric cancer patients. In addition, the reduced expression of ZHX2 was associated with a favorable overall survival rate in gastric cancer patients who only received surgery, but not in the patients who received additional therapies [31]. Taken together, these data suggest that ZHX2 and ZHX3 act as oncogenes in gastric cancer. 

The expression levels of ZHX2 in nature killer (NK) cells have shown an important clinical significance [65]. Kaplan–Meier analysis of overall survival from a TCGA dataset showed that a lower ZHX2 expression level in NK cells was associated with better prognosis in HCC patients. The genetical deleting ZHX2 gene improves IL-15-mediated NK cell activity, maturation, and cell viability in vitro, resulting in better antitumor immunity. Therapeutically, the transfer of ZHX2-deficient NK cells inhibited hepatoma homograft tumor growth and metastasis. These studies reveal a novel regulatory function of ZHX2 in NK cell maturation and its therapeutic potential by enhancing NK cell-mediated cancer surveillance [65].

### 2.3. ZHX3 and Cancer 

**Figure 3:**
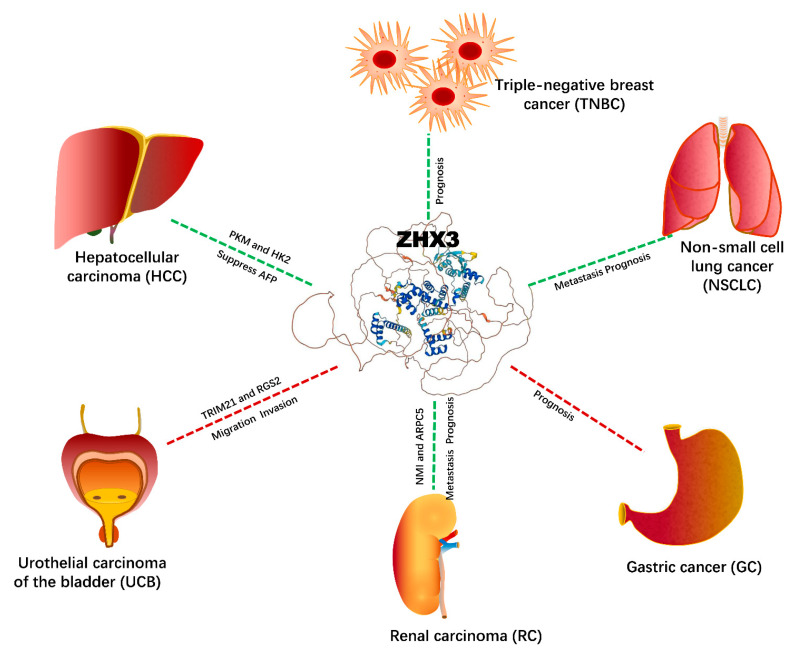
ZHX3 and cancers: biological functions and potential signaling networking.

The biological functions of ZHX3 in cancer have not been well characterized, but limited studies have shown that ZHX3 is a tumor suppressor in HCC, non-small cell lung cancer, breast cancer, and renal cancer (Table 3). It has also been reported that an increased ZHX3 expression is associated with the progression of bladder carcinoma and gastric cancer, indicating that ZHX3 is involved in oncogenic programs (Table 3). The clinical significance of ZHX3 and the underlying potential mechanism are summarized in Figure 3.

ZHX3 was first identified as a suppressor of the AFP gene in HCC and was a good candidate for the tumor suppressor present at 16q22 [16]. As with ZHX2, ZHX3 is expressed at very low levels in HCC cells compared with rat hepatocytes. ZHX3 repressed the transcription of the luciferase reporter gene that was fused to the promoters of PKM and HK2 [16]. This suggests that the loss of expression of ZHX3 might be a critical factor during hepatocellular carcinogenesis.

ZHX3 expression was also remarkably decreased in non-small cell lung cancer (NSCLC) in comparison to the adjacent non-tumor tissues [66]. Interestingly, a lower ZHX3 expression in the tumor had a significantly greater risk of lymph node metastasis and was associated with a poorer survival time. Therefore, ZHX3 was an independent factor affecting metastasis and in predicting the 5-year overall survival rate among NSCLC patients.

A recent study on renal carcinoma showed a tumor-suppressive role for ZHX3 [18]. As seen with ZHX1, ZHX3 showed a lower expression in cancers, and was associated with a high-risk of lymph node metastasis and worse outcomes. Further analysis of mRNA co-occurrence using cBioPortal data (www.cbioportal.org) showed opposite relationships between the expressions of ZHX1 and ZHX3 and some well-known oncogenes [18]. For instance, ZHX3 expression was negatively associated with the expressions of N-myc, STAT Interactor (NMI), and actin-related protein 2/3 complex subunit 5 (ARPC5). Both NMI and ARPC5 play critical roles in initiating cancer formation and facilitating cancer cell proliferation, migration, and metastasis [67,68]. 

In breast cancer, a negative ZHX3 expression was correlated with lymph node metastasis, poor differentiation, advanced tumor stage, and positive estrogen receptor expression [19]. The immunohistochemical (IHC) staining analysis showed that the patients with decreased ZHX3 protein levels had a poor prognosis. The increased ZHX3 expression was associated with good outcomes in breast cancer patients, indicating that ZHX3 might act as a prognostic biomarker for breast cancer patients [19].

ZHX3 expression can also be used as a prognostic indicator for gastric cancer [31]. IHC staining confirmed that gastric cancer patients with a high ZHX3 expression have a worse prognosis [31].

In a recent study [20], ZHX3 was first screened in a urothelial carcinoma of the bladder (UCB) as a critical oncogenic factor associated with poor prognosis—this was based from The Cancer Genome Atlas dataset and a large cohort of UCB clinical samples. ZHX3 promoted the migration and invasion of UCB cell in vitro and in vivo. Mechanistically, ZHX3 repressed the expression of regulator of G protein signaling 2 (RGS2). Meanwhile, as a target, ZHX3 was regulated by tripartite motif 21 (TRIM21), which mediates its ubiquitination and subsequent degradation. These results indicate that ZHX3 is an oncogene and has a therapeutic potential for UCB [20].

### 2.4. ZHXs and Cancer Patient Survival 

**Figure 4:**
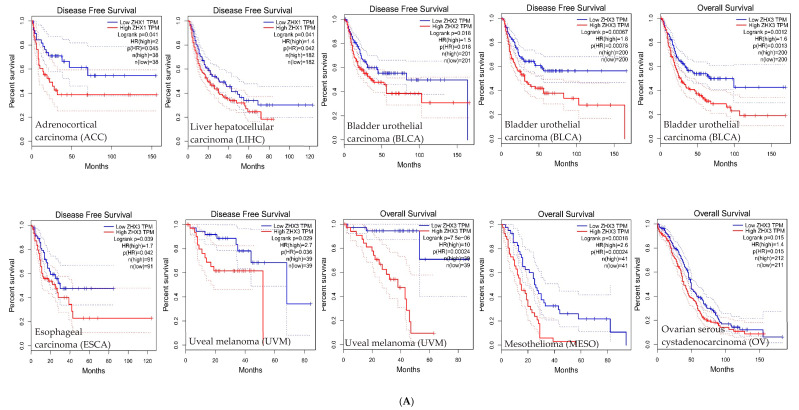

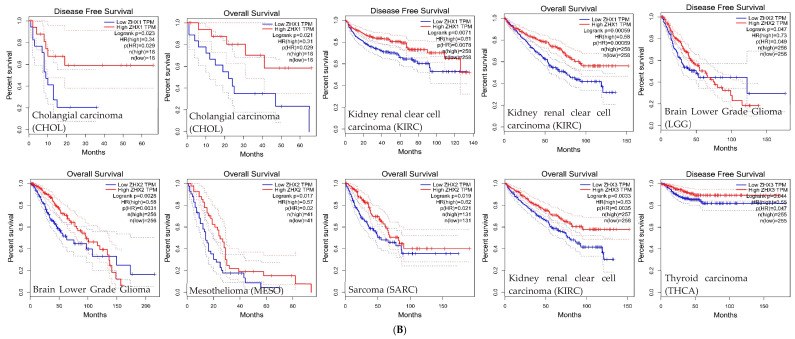
ZHX1, ZHX2, and ZHX3 gene expression level and survival analysis in 12 types of cancers. These panels were generated using the GEPIA database (http://gepia.cancer-pku.cn/) on 06/02/2022. (**A**): Patients with low ZHX1, ZHX2, or ZHX3 expressions have better OS or DFS. The words in red stood for ZHX1, ZHX2, and ZHX3 high expressions, the words in blue stood for ZHX1, ZHX2, and ZHX3 low expressions. (**B**): Patients with high ZHX1, ZHX2, or ZHX3 expressions have better OS or DFS. GEPIA performs overall survival (OS) or disease-free survival (DFS) analysis based on ZHX1, ZHX2, and ZHX3 gene expressions. The cox proportional hazard ratio and 95% confidence interval information can also be included in the survival plot. “Median” was used in group cutoff. Cutoff-high (%) indicates that samples with expression levels higher than this threshold are considered as the high-expression cohort and samples with expression levels lower than this threshold are considered the low-expression cohort. Cancer types of abbreviations: ACC, adrenocortical carcinoma; BLCA, bladder urothelial carcinoma; CHOL, cholangial carcinoma; ESCA, esophageal carcinoma; KIRC, kidney renal clear cell carcinoma; LGG, brain lower grade glioma; LIHC, liver hepatocellular carcinoma; MESO, mesothelioma; OV, ovarian serous cystadenocarcinoma; SARC, sarcoma; THCA, thyroid carcinnooma; UVM, uveal melanoma.

The association between ZHX family member expression levels and the survival time in multiple types of cancers were refined by us using the comprehensive datasets from the GEPIA (Gene Expression Profiling Interactive Analysis, http://gepia.cancer-pku.cn/). We selected overall survival (OS) or disease-free survival (DFS) as survival time indicators. Figure 4A shows that the cancer types with a low ZHX1, ZHX2, or ZHX3 expression have better OS or DFS, including ZHX1 in adrenocortical carcinoma and liver hepatocellular carcinoma, ZHX2 in bladder urothelial carcinoma, and ZHX3 in bladder urothelial carcinoma, esophageal carcinoma, uveal melanoma, mesothelioma, and ovarian serous cystadenocarcinoma. In contrast, the types of cancers with high ZHX1, ZHX2, or ZHX3 expressions have better OS or DFS, as shown in Figure 4B, including ZHX1 in cholangial carcinoma and kidney renal clear cell carcinoma, ZHX2 in brain lower grade glioma, mesothelioma and sarcoma, and ZHX3 in renal clear cell carcinoma and thyroid carcinoma. These results again have proven the roles of the ZHX genes as a double-edged sword in cancer patient survival.

## 3. Conclusions and Perspectives

**Figure 5:**
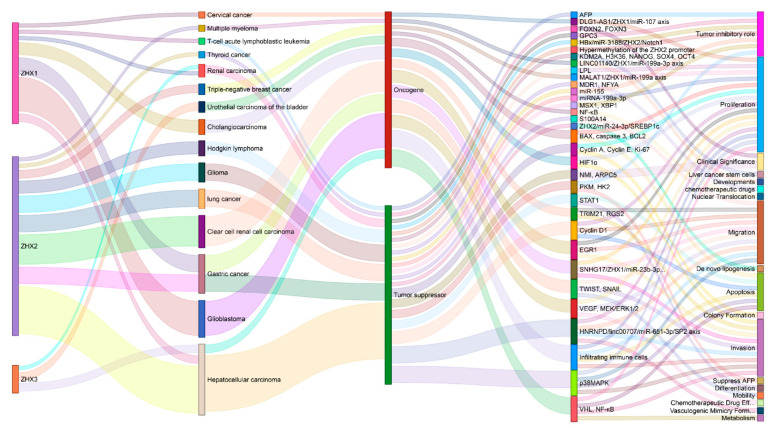
A comprehensive illustration of ZHXs as a double-edged sword in cancers: an oncogene or tumor suppressor, underlying mechanisms, and biological functions.

Currently, studies on the ZHX family members in cancer have demonstrated tumor-suppressive and oncogenic roles in carcinogenesis and progression by power BI tools (Figure 5). These opposing effects are also seen in the roles of ZHXs in the prediction of outcomes and responses to therapy. These differences are most likely specific to cancer type. However, the studies are still limited to a few cancer types. The biological functions of ZHXs in cancers of the respiratory system, gastrointestinal tract, head and neck, and reproductive system are largely unknown, and thus, require further investigation. Moreover, neither ZHX-related signaling pathways nor upstream and downstream regulators in carcinogenesis and therapy are clear, and thus, need further elucidation.

## Figures and Tables

**Table 1:** Studies on ZHX1 expression, function, clinical significance, and molecular mechanism.

ZHX Family Member	Expression Levels	Functions	Cancer Types	Clinical Significance	Associated Effects	Involved Molecules	References
ZHX1	Increased	Oncogene	Glioblastoma	ZHX1 overexpression was associated with short overall survival.	Facilitated proliferation, mobility, migration, and invasion.	TWIST and SNAIL	[23]
ZHX1	Increased	Oncogene	Glioblastoma		Promoted proliferation and progression.	LncRNA MALAT1/ZHX1/miR-199a axis	[24]
ZHX1	Increased	Oncogene	Glioblastoma		Promoted proliferation, migration, and invasion.	LncRNA SNHG17/ZHX1/miR-23b-3p axis	[25]
ZHX1	Increased	Oncogene	Glioblastoma		Promoted glioma development.	LncRNA LINC01140/ZHX1/miR-199a-3p axis	[26]
ZHX1	Increased	Oncogene	Cervical cancer	Cervical cancer patients with high DLG1-AS1 expression had worse prognosis.	Promoted proliferation of cervical cancer cells.	LncRNA DLG1-AS1/ZHX1/miR-107 axis	[27]
ZHX1	Increased	Oncogene	Cholangiocarcinoma		Promoted proliferation, migration, and invasion of cholangiocarcinoma cells.	Early growth response 1 (EGR1)	[28]
ZHX1	Decreased	Tumor suppressor	Gastric cancer		Promoted apoptosis, and inhibited cell proliferation and migration.	Cyclin D1	[29]
ZHX1	Decreased	Tumor suppressor	Gastric cancer		Inhibited cell proliferation.	miRNA-199a-3p	[30]
ZHX1	Decreased	Tumor suppressor	Gastric cancer	Increased ZHX1 expression was associated with better OS in patients with gastric cancer, without distant metastasis, received surgery, and poorly differentiated.			[31]
ZHX1	Decreased	Tumor suppressor	Renal cancers	Reduction in ZHX1 mRNA expression was associated with the progression of clear cell renal cancers and poorer survival.			[18]
ZHX1	Decreased	Tumor suppressor	Hepatocellular carcinoma		Inhibited the proliferation of HCC SMMC-7721 cells.		[32]
ZHX1	Increased	Oncogene	Hepatocellular carcinoma		Promoted cell growth and inhibited apoptosis.	BAX, caspase 3 and BCL2	[33]
ZHX1 and ZHX2	Decreased	Tumor suppressor	Chronic lymphocytic leukemia (CLL)	CLL patient with lower expression level of ZHX1 and ZHX2 had worse prognosis.			[34]
ZHX1	Decreased	Tumor suppressor	T-cell acute lymphoblastic leukemia (T-ALL)			FOXN2 and FOXN3	[35]
ZHX1	Increased	Oncogene	Hodgkin lymphoma (HL)				[36]

**Table 2:** Studies on ZHX2 expression, function, clinical significance, and molecular mechanism.

ZHX Family Member	Expression Levels	Functions	Cancer Types	Clinical Significance	Associated Effects	Molecular Mechanisms	References
ZHX2	Decreased	Tumor suppressor	Hepatocellular carcinoma (HCC)		Inhibited HCC cell proliferation and colony formation in vitro and xenograft growth in nude mice.	Cyclin A, Cyclin E and Ki-67	[37]
ZHX2	Decreased	Tumor suppressor	HCC			Hypermethylation of the ZHX2 promoter	[38]
ZHX2	Decreased	Tumor suppressor	HCC		ZHX2 expression in HCC was inversely correlated with serum AFP levels in HCC patients.		[39]
ZHX2	Decreased	Tumor suppressor	HCC			Repression of AFP	[40]
ZHX2	Decreased	Tumor suppressor	HCC			Repression of PKM, HK2	[16]
ZHX2	Decreased	Tumor suppressor	HCC			Repression of GPC3	[41]
ZHX2	Decreased	Tumor suppressor	HBV-related HCC			HBx/miR-3188/ZHX2/Notch1 signaling pathway	[42]
ZHX2	Decreased	Tumor suppressor	HBV-related HCC			miR-155	[43]
ZHX2	Decreased	Tumor suppressor	Lipogenesis-related HCC		Inhibited de novo lipogenesis in HCC cells and HCC progression.	ZHX2/miR-24–3p/SREBP1c	[44]
ZHX2	Decreased	Tumor suppressor	NAFLD-associated HCC		Inhibited HCC cell proliferation, xenograft tumor growth, lipid deposition.	LPL	[45]
ZHX2	Decreased	Tumor suppressor	Liver cancer stem cells (CSCs)	The lower ZHX2 expression and higher KDM2A were associated with poorer survival of patients.	Restricted tumor initiation, self-renewal and sorafenib-resistance of hepatic CSCs.	KDM2A, H3K36, NANOG, SOX4 and OCT4	[46]
ZHX2	Increased	Oncogene	Hepatocellular carcinoma (HCC)	Upregulation of ZHX2 was correlated with poor differentiation and cancer metastasis.			[47]
ZHX2	Decreased	Tumor suppressor	Liver tumor		Enhances the cytotoxicity of chemotherapeutic drugs.	Repressing multidrug resistance 1 (MDR1) via an interaction with NFYA	[48]
ZHX2	Decreased	Tumor suppressor	lung cancer		Suppressed cells proliferation, migration and invasion and promoted apoptosis.	p38MAPK signaling pathway	[49]
ZHX2	Decreased	Tumor suppressor	Glioma		Inhibited proliferation, migration, invasion and VM formation.	HNRNPD/linc00707/miR-651–3p/SP2 axis	[50]
ZHX2	Decreased	Tumor suppressor	Thyroid cancer	Decreased ZHX2 expression was correlated with poor prognosis of thyroid cancer patients.	ZHX2 knockdown significantly promoted the migration of thyroid cancer cells.	S100A14	[51]
ZHX2	Decreased	Tumor suppressor	Multiple myeloma (MM)	Increased ZHX2 expression with improved response to high-dose chemotherapy in multiple myeloma.			[52,53]
ZHX2	Decreased	Tumor suppressor	Multiple myeloma (MM)	Low expression of ZHX2 is associated with poor outcome in multiple myeloma.			[54]
ZHX2	Increased	Oncogene	Multiple myeloma (MM)	Multiple myeloma patients with higher ZHX2 expression showed poorer clinical outcomes of.	Knockdown of ZHX2 significantly enhanced the sensitivity of MM cells to Bortezomib (BTZ), inhibited nuclear translocation of NF-κB.	NF-κB.	[55]
ZHX2	Decreased	Tumor suppressor	Hodgkin lymphoma		Knockdown of ZHX2 led to inhibition of genes regulating differentiation and apoptosis.	STAT1	[56]
ZHX2	Decreased	Tumor suppressor	Hodgkin lymphoma			MSX1 and XBP1	[57]
ZHX2	Increased	Oncogene	Clear cell renal cell carcinoma (ccRCC)		Depletion of ZHX2 inhibited VHL-deficient ccRCC cell growth and caused decreased expression of multiple genes linked with anti-apoptosis, cell proliferation, invasion/metastasis, and metabolism.	VHL, NF-κB	[21]
ZHX2	Increased	Oncogene	Clear cell renal cell carcinoma (ccRCC)		ZHX2 drove cell growth, migration and induced Sunitinib resistance by regulating self-protective autophagy.	VEGF, MEK/ERK1/2 signaling pathway	[58]
ZHX2	Increased	Oncogene	Triple-negative breast cancer (TNBC)		Depletion of ZHX2 inhibited TNBC cell growth and invasion in vitro, orthotopic tumor growth, and spontaneous lung metastasis in vivo.	HIF1α, H3K4me3 and H3K27ac; AP2B1, COX20, KDM3A, or PTGES3L; R491, R581, and R674	[59]
ZHX2 and ZHX3	Decreased	Tumor suppressor	Triple-negative breast cancer (TNBC)	High mRNA expression of ZHX2 and ZHX3 was significantly correlated with better OS of breast cancer patients.			[19]
ZHX2	Increased	Oncogene	Gastric cancer (GC)	Upregulation of ZHX2 predicted poor prognosis in GC.	Promotes proliferation, invasion, migration, and inhibits cell apoptosis.	Immune infiltrating	[60]
ZHX2 and ZHX3	Increased	Oncogene	Gastric cancer (GC)	Decreased mRNA expression of ZHX2 and ZHX3 was correlated with better rates of OS in patients with gastric cancer.			[31]

**Table 3:** Studies on ZHX3 expression, function, clinical significance, and molecular mechanism.

ZHX Family Member	Expression Levels	Functions	Cancer Types	Clinical Significance	Associated Effects	Molecular Mechanisms	References
ZHX3	Decreased	Tumor suppressor	HCC	ZHX3 was first identified as a suppressor of the AFP gene.		PKM and HK2	[16]
ZHX3	Decreased	Tumor suppressor	Non-small cell lung cancer (NSCLC)	Lower ZHX3 expression in the tumor had a significant greater risk of lymph node metastasis and was associated with poorer survival time.			[66]
ZHX1 and ZHX3	Decreased	Tumor suppressor	Renal carcinoma	A lower ZHX3 expression in cancers was correlated with a high risk of lymph node metastasis and worse outcomes.		NMI and ARPC5	[18]
ZHX3	Decreased	Tumor suppressor	Breast cancer	Patients with decreased ZHX3 protein levels had poorer outcomes.			[19]
ZHX3	Increased	Oncogene	Gastric cancer	Over-expression of ZHX3 was associated with worse OS.			[31]
ZHX3	Increased	Oncogene	Urothelial carcinoma of the bladder (UCB)		Promote the migration and invasion capacities of UCB cells both in vitro and in vivo.	TRIM21 and RGS2	[20]

## Data Availability

Not applicable.
